# Supplementary material for: Assessing the Optimal Regimen: A Systematic Review and Network Meta-Analysis of the Efficacy and Safety of Long-Acting Granulocyte Colony-Stimulating Factors in Patients with Breast Cancer
Source: Cancers (Basel). 2023 Jul 19;15(14):3675. doi: 10.3390/cancers15143675 (PMC10378237; doi:10.3390/cancers15143675)
Supplement: Supplementary file 1 [file cancers-15-03675-s001.zip › cancers-2392214-supplementary.pdf]

Table of Contents

1. Graph of Bias 3
Drug-based Analysis 5
2. Duration of Severe Neutropenia 5
2.1. Result of Heterogeneity and Consistency Analysis 5
2.2. Forest Plots of the Network Meta-analysis for Duration of Severe Neutropenia..9
2.3. Summary of confidence in network estimates 10
3. Absolute Neutrophil Count Recovery Time 12
3.1. Result of Heterogeneity and Consistency Analysis 12
3.2. Forest plots of the network meta-analysis for Absolute Neutrophil Count Recovery Time 14
3.3. Summary of confidence in network estimates 16
4. Incidence of Severe Neutropenia 17
4.1. Result of Heterogeneity and Consistency Analysis 17
4.2. Forest plots of the network meta-analysis for Incidence of Severe Neutropenia..18
4.3. Summary of confidence in network estimates 19
5. Incidence of Febrile Neutropenia...20
5.1. Result of Heterogeneity and Consistency Analysis 20
5.2. Summary of confidence in network estimates 23
6. Grade 3-4 Adverse Event 24
6.1. Result of Heterogeneity and Consistency Analysis 24
6.2. Forest plots of the network meta-analysis for Grade 3-4 Adverse Event 25
6.3. Summary of confidence in network estimates 26
7. SUCRA results (percentages) for other drugs of pegfilgrastim and its biosimilar...27
Dose-based Analysis 28
8. Duration of Severe Neutropenia 28
8.1. Result of Heterogeneity and Consistency Analysis 28
8.2. Forest plots of the network meta-analysis for Duration of Severe Neutropenia..29
8.3. Summary of confidence in network estimates 30
9. Absolute Neutrophil Count Recovery Time 31
9.1. Result of Heterogeneity and Consistency Analysis 31
9.2. Forest plots of the network meta-analysis for Absolute Neutrophil Count Recovery Time 32
9.3. Summary of confidence in network estimates 33
10. Incidence of Severe Neutropenia 34
10.1. Result of Heterogeneity and Consistency Analysis 34
10.2. Forest plots of the network meta-analysis for Incidence of Severe Neutropenia..35
10.3. Summary of confidence in network estimates 36

11. Incidence of Febrile Neutropenia 37
11.1. Result of Heterogeneity and Consistency Analysis 37
11.2. Forest plots of the network meta-analysis for Incidence of Febrile Neutropenia 38
11.3. Summary of confidence in network estimates 39
12. Grade 3-4 Adverse Event 40
12.1. Result of Heterogeneity and Consistency Analysis 40
12.2. Forest plots of the network meta-analysis for Grade 3-4 Adverse Event 42
12.3. Summary of confidence in network estimates 43
13. Search Strategy 44
14. PRISMA checklist 45

1. Graph of Bias

Figure. S1. Risk of Bias Graph. Review authors' judgements about each risk of bias item presented as percentages across all included trials.

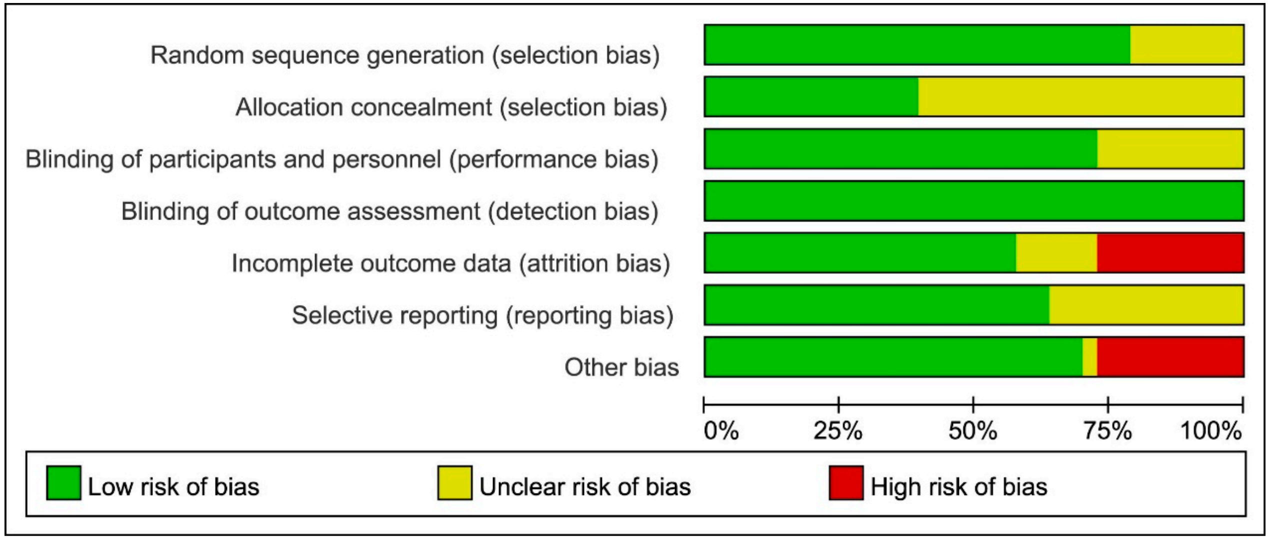

Figure. S2. Risk of Bias Graph. Review authors' judgements about risk of bias in each trial.

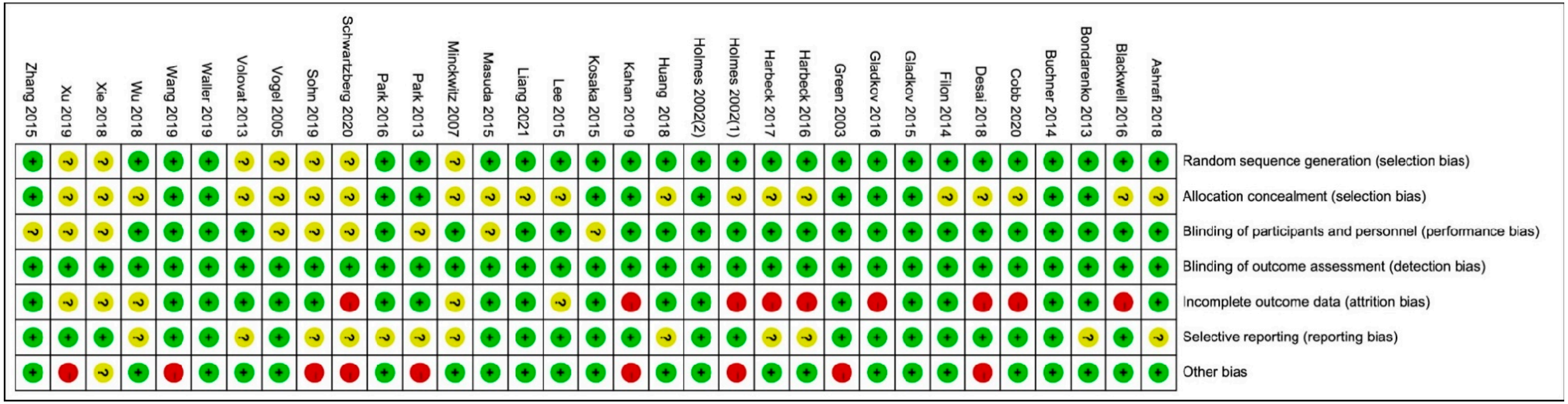

Drug-based Analysis

2. Duration of Severe Neutropenia

2.1. Result of Heterogeneity and Consistency Analysis

```
> gelman.diag(DSNrslt)
Potential scale reduction factors:

       Point est. Upper C.I.
d.2.1           1          1
d.2.10          1          1
d.2.11          1          1
d.2.12          1          1
d.2.13          1          1
d.2.14          1          1
d.2.17          1          1
d.2.18          1          1
d.2.23          1          1
d.2.26          1          1
d.2.27          1          1
d.2.28          1          1
d.2.3           1          1
d.2.6           1          1
d.2.9           1          1
d.6.16          1          1
d.6.19          1          1
d.6.21          1          1
d.6.22          1          1
d.6.24          1          1
d.6.25          1          1
d.6.29          1          1
d.6.32          1          1
d.6.33          1          1
d.6.4           1          1
d.6.5           1          1
d.6.7           1          1
d.6.8           1          1
```

```
Multivariate psrf
1
```

Analysis of heterogeneity

========================

Per-comparison I-squared:

----

	t1	t2	i2.pair	i2.cons	incons.p
1	1	2	52.11217	52.112198	NA
2	10	2	0.00000	0.000000	NA
3	11	12	NA	NA	NA
4	11	13	NA	NA	NA
5	11	2	NA	NA	NA
6	12	13	NA	NA	NA
7	12	2	NA	NA	NA
8	13	2	0.00000	0.000000	NA
9	14	2	NA	NA	NA
10	16	19	NA	NA	NA
11	16	6	19.16658	19.907131	NA
12	17	2	0.00000	0.620683	NA
13	17	3	NA	NA	NA
14	17	9	26.42928	32.027401	NA
15	18	2	NA	NA	NA
16	19	6	NA	NA	NA
17	2	23	NA	NA	NA
18	2	26	NA	NA	NA
19	2	27	NA	NA	NA
20	2	28	NA	NA	NA
21	2	3	NA	NA	NA
22	2	6	NA	NA	NA
23	2	9	0.00000	0.000000	NA
24	21	22	NA	NA	NA
25	21	24	0.00000	0.000000	NA
26	21	25	NA	NA	NA
27	21	6	0.00000	0.000000	NA
28	22	24	NA	NA	NA
29	22	6	NA	NA	NA
30	24	25	NA	NA	NA
31	24	6	0.00000	0.000000	NA
32	25	6	NA	NA	NA

```
33 26 27        NA         NA        NA
34 29  6        NA         NA        NA
35  3  9        NA         NA        NA
36 32 33        NA         NA        NA
37 32  6        NA         NA        NA
38 33  6        NA         NA        NA
39  4  5        NA         NA        NA
40  4  6        NA         NA        NA
41  5  6        NA         NA        NA
42  6  7        NA         NA        NA
43  6  8        NA         NA        NA
44  7  8        NA         NA        NA

Global I-squared:
-------------------------


  i2.pair i2.cons
1       0       0

-- Model fit (residual deviance):


     Dbar        pD       DIC
 62.86689  52.97428 115.84116

64 data points, ratio 0.9823, I^2 = 0%
```

2.2. Forest Plots of the Network Meta-analysis for Duration of Severe Neutropenia

Figure. S3. Forest plots of the network meta-analysis for the duration of severe neutropenia comparing different LA-G-CSFs with reference drug of pegfilgrastim 6 mg. The bars indicate mean difference (MD) and 95% credibility intervals (CrIs). LA-G-CSF = long-acting granulocyte colony-stimulating factor, SA-G-CSF = short-acting G-CSF.

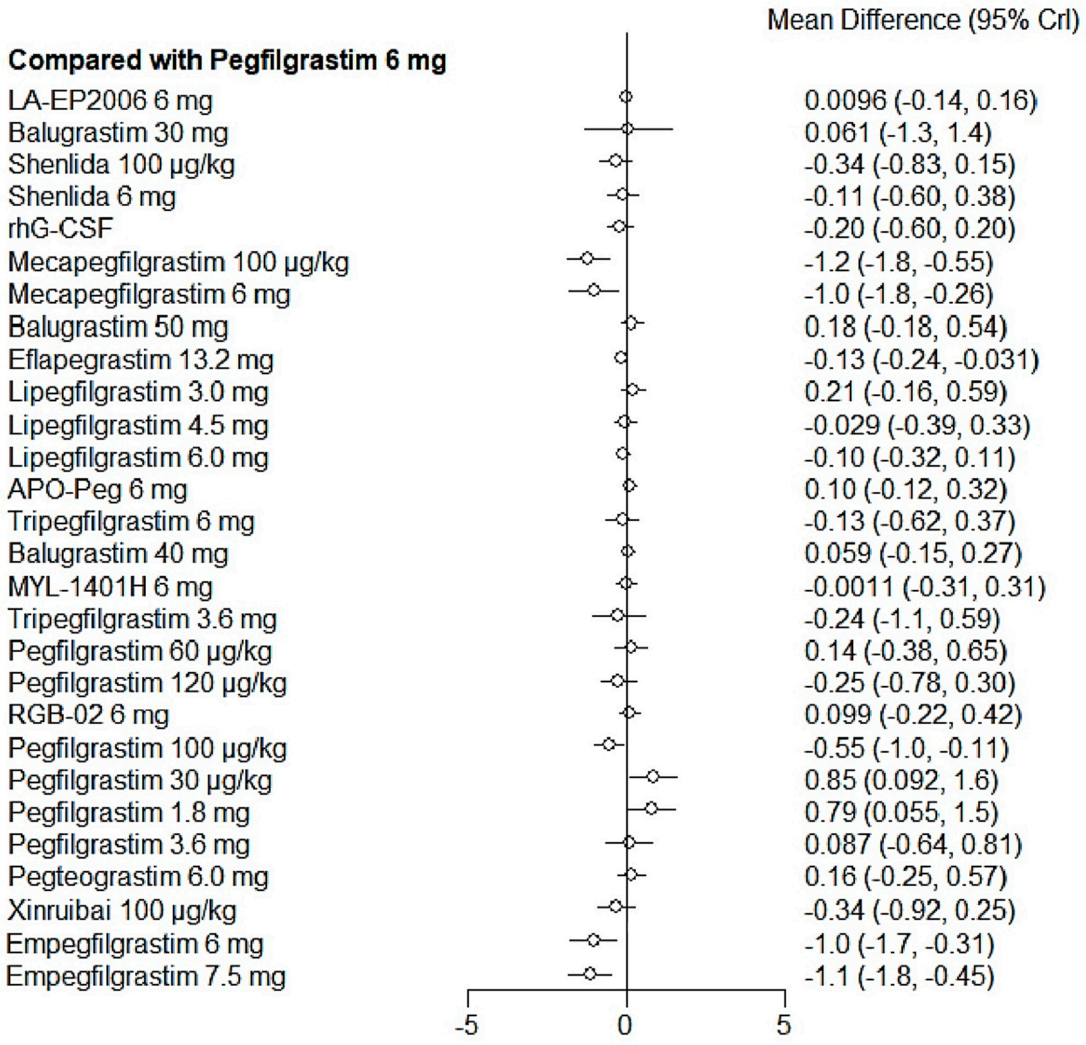

2.3. Summary of confidence in network estimates

Figure. S4. CINeMA rankings comparing different LA-G-CSFs with SA-G-CSF (A) and with pegfilgrastim 6 mg (B) of duration of SN in drug-based analysis.

Comparison	Number of studies	Within-study bias	Reporting bias	Indirectness	Imprecision	Heterogeneity	Incoherence	Confidence rating
Empegfilgrastim 6 mg:rhG-CSF	1	No concerns	Low risk	No concerns	No concerns	No concerns	No concerns	High
Empegfilgrastim 7.5 mg:rhG-CSF	1	No concerns	Low risk	No concerns	No concerns	No concerns	No concerns	High
Mecapegfilgrastim 100 ug/kg:rhG-CSF	1	Some concerns	Low risk	No concerns	No concerns	No concerns	No concerns	High
Mecapegfilgrastim 6 mg:rhG-CSF	1	Some concerns	Low risk	No concerns	No concerns	No concerns	No concerns	High
Pegfilgrastim 100 ug/kg:rhG-CSF	3	No concerns	Low risk	No concerns	No concerns	No concerns	No concerns	High
Pegfilgrastim 120 ug/kg:rhG-CSF	1	No concerns	Low risk	No concerns	No concerns	No concerns	No concerns	High
Pegfilgrastim 30 ug/kg:rhG-CSF	1	Some concerns	Low risk	No concerns	No concerns	No concerns	No concerns	High
Pegfilgrastim 6 mg:rhG-CSF	1	Some concerns	Low risk	No concerns	No concerns	No concerns	No concerns	High
Pegfilgrastim 60 ug/kg:rhG-CSF	2	Some concerns	Low risk	No concerns	No concerns	No concerns	No concerns	High
Shenlida 100 ug/kg:rhG-CSF	1	Some concerns	Low risk	No concerns	No concerns	No concerns	No concerns	High
Shenlida 6 mg:rhG-CSF	1	Some concerns	Low risk	No concerns	No concerns	No concerns	No concerns	High
Tripegfilgrastim 3.6 mg:rhG-CSF	1	No concerns	Low risk	No concerns	No concerns	No concerns	No concerns	High
Tripegfilgrastim 6 mg:rhG-CSF	2	No concerns	Low risk	No concerns	No concerns	No concerns	No concerns	High
Xinruibai 100 ug/kg:rhG-CSF	1	No concerns	Low risk	No concerns	No concerns	No concerns	No concerns	High
APO-Peg 6 mg:rhG-CSF	0	Major concerns	Low risk	No concerns	No concerns	No concerns	No concerns	Moderate
Balugrastim 30 mg:rhG-CSF	0	Some concerns	Low risk	No concerns	No concerns	Some concerns	No concerns	Moderate
Balugrastim 40 mg:rhG-CSF	0	Some concerns	Low risk	No concerns	No concerns	No concerns	No concerns	High
Balugrastim 50 mg:rhG-CSF	0	Some concerns	Low risk	No concerns	No concerns	No concerns	No concerns	High
Eflapegrastim 13.2 mg:rhG-CSF	0	Some concerns	Low risk	No concerns	No concerns	No concerns	No concerns	High
LA-EP2006 6 mg:rhG-CSF	0	Some concerns	Low risk	No concerns	No concerns	No concerns	No concerns	High
Lipegfilgrastim 3 mg:rhG-CSF	0	No concerns	Low risk	No concerns	No concerns	No concerns	No concerns	High
Lipegfilgrastim 4.5 mg:rhG-CSF	0	No concerns	Low risk	No concerns	No concerns	No concerns	No concerns	High
Lipegfilgrastim 6 mg:rhG-CSF	0	No concerns	Low risk	No concerns	No concerns	No concerns	No concerns	High
MYL-1401H 6 mg:rhG-CSF	0	Some concerns	Low risk	No concerns	No concerns	No concerns	No concerns	High
Pegfilgrastim 1.8 mg:rhG-CSF	0	No concerns	Low risk	No concerns	No concerns	No concerns	No concerns	High
Pegfilgrastim 3.6 mg:rhG-CSF	0	No concerns	Low risk	No concerns	No concerns	No concerns	No concerns	High
Pegteograstim 6.0 mg:rhG-CSF	0	Some concerns	Low risk	No concerns	No concerns	No concerns	No concerns	High
RGB-02 6 mg:rhG-CSF	0	Major concerns	Low risk	No concerns	No concerns	No concerns	No concerns	Moderate

Comparison	Number of studies	Within-study bias	Reporting bias	Indirectness	Imprecision	Heterogeneity	Incoherence	Confidence rating
APO-Peg 6 mg:Pegfilgrastim 6 mg	1	Major concerns	Low risk	No concerns	No concerns	No concerns	No concerns	Moderate
Balugrastim 30 mg:Pegfilgrastim 6 mg	1	No concerns	Low risk	No concerns	No concerns	No concerns	No concerns	High
Balugrastim 40 mg:Pegfilgrastim 6 mg	3	No concerns	Low risk	No concerns	No concerns	No concerns	No concerns	High
Balugrastim 50 mg:Pegfilgrastim 6 mg	2	Some concerns	Low risk	No concerns	No concerns	No concerns	No concerns	High
Eflapegrastim 13.2 mg:Pegfilgrastim 6 mg	2	Major concerns	Low risk	No concerns	No concerns	No concerns	No concerns	Moderate
LA-EP2006 6 mg:Pegfilgrastim 6 mg	2	Some concerns	Low risk	No concerns	No concerns	No concerns	No concerns	High
Lipegfilgrastim 3 mg:Pegfilgrastim 6 mg	1	No concerns	Low risk	No concerns	No concerns	No concerns	No concerns	High
Lipegfilgrastim 4.5 mg:Pegfilgrastim 6 mg	1	No concerns	Low risk	No concerns	No concerns	No concerns	No concerns	High
Lipegfilgrastim 6 mg:Pegfilgrastim 6 mg	2	No concerns	Low risk	No concerns	No concerns	No concerns	No concerns	High
MYL-1401H 6 mg:Pegfilgrastim 6 mg	1	No concerns	Low risk	No concerns	No concerns	No concerns	No concerns	High
Pegfilgrastim 1.8 mg:Pegfilgrastim 6 mg	1	No concerns	Low risk	No concerns	No concerns	No concerns	No concerns	High
Pegfilgrastim 3.6 mg:Pegfilgrastim 6 mg	1	No concerns	Low risk	No concerns	No concerns	No concerns	No concerns	High
Pegfilgrastim 6 mg:Pegteograstim 6.0 mg	1	No concerns	Low risk	No concerns	No concerns	No concerns	No concerns	High
Pegfilgrastim 6 mg:RGB-02 6 mg	1	Major concerns	Low risk	No concerns	No concerns	No concerns	No concerns	Moderate
Pegfilgrastim 6 mg:rhG-CSF	1	Some concerns	Low risk	No concerns	No concerns	No concerns	No concerns	High
Empegfilgrastim 6 mg:Pegfilgrastim 6 mg	0	No concerns	Low risk	No concerns	No concerns	No concerns	No concerns	High
Empegfilgrastim 7.5 mg:Pegfilgrastim 6 mg	0	No concerns	Low risk	No concerns	No concerns	No concerns	No concerns	High
Mecapegfilgrastim 100 ug/kg:Pegfilgrastim 6 mg	0	Some concerns	Low risk	No concerns	No concerns	No concerns	No concerns	High
Mecapegfilgrastim 6 mg:Pegfilgrastim 6 mg	0	Some concerns	Low risk	No concerns	No concerns	Some concerns	No concerns	High
Pegfilgrastim 100 ug/kg:Pegfilgrastim 6 mg	0	Some concerns	Low risk	No concerns	No concerns	No concerns	No concerns	High
Pegfilgrastim 120 ug/kg:Pegfilgrastim 6 mg	0	Some concerns	Low risk	No concerns	No concerns	No concerns	No concerns	High
Pegfilgrastim 30 ug/kg:Pegfilgrastim 6 mg	0	Some concerns	Low risk	No concerns	No concerns	Some concerns	No concerns	High
Pegfilgrastim 60 ug/kg:Pegfilgrastim 6 mg	0	Some concerns	Low risk	No concerns	No concerns	No concerns	No concerns	High
Pegfilgrastim 6 mg:Shenlida 100 ug/kg	0	Some concerns	Low risk	No concerns	No concerns	No concerns	No concerns	High
Pegfilgrastim 6 mg:Shenlida 6 mg	0	Some concerns	Low risk	No concerns	No concerns	No concerns	No concerns	High
Pegfilgrastim 6 mg:Tripegfilgrastim 3.6 mg	0	No concerns	Low risk	No concerns	No concerns	No concerns	No concerns	High
Pegfilgrastim 6 mg:Tripegfilgrastim 6 mg	0	No concerns	Low risk	No concerns	No concerns	No concerns	No concerns	High
Pegfilgrastim 6 mg:Xinruibai 100 ug/kg	0	Some concerns	Low risk	No concerns	No concerns	No concerns	No concerns	High

3. Absolute Neutrophil Count Recovery Time

3.1. Result of Heterogeneity and Consistency Analysis

```
> gelman.diag(DSNrslt)
Potential scale reduction factors:

       Point est. Upper C.I.
d.2.1           1          1
d.2.10          1          1
d.2.11          1          1
d.2.12          1          1
d.2.13          1          1
d.2.14          1          1
d.2.17          1          1
d.2.18          1          1
d.2.23          1          1
d.2.26          1          1
d.2.27          1          1
d.2.28          1          1
d.2.3           1          1
d.2.6           1          1
d.2.9           1          1
d.6.16          1          1
d.6.19          1          1
d.6.21          1          1
d.6.22          1          1
d.6.24          1          1
d.6.25          1          1
d.6.29          1          1
d.6.32          1          1
d.6.33          1          1
d.6.4           1          1
d.6.5           1          1
d.6.7           1          1
d.6.8           1          1

Multivariate psrf
1
```

```
Analysis of heterogeneity
=========================

Per-comparison I-squared:
-------------------------

   t1 t2  i2.pair  i2.cons incons.p
1   1  2       NA       NA       NA
2  10  2       NA       NA       NA
3  13  2       NA       NA       NA
4  14  2       NA       NA       NA
5  16 19       NA       NA       NA
6  16  6  0.00000  0.00000       NA
7  17  2 58.97176 60.22930       NA
8  17  3       NA       NA       NA
9  17  9 15.06937 40.52076       NA
10 18  2       NA       NA       NA
11 19  6       NA       NA       NA
12  2 21       NA       NA       NA
13  2 22       NA       NA       NA
14  2 23       NA       NA       NA
15  2 28       NA       NA       NA
16  2  3       NA       NA       NA
17  2  6       NA       NA       NA
18  2  9  0.00000  0.00000       NA
19 21 22       NA       NA       NA
20 21 25       NA       NA       NA
21 21  6  0.00000  0.00000       NA
22 22  6       NA       NA       NA
23 25  6       NA       NA       NA
24 29  6       NA       NA       NA
25  3  9       NA       NA       NA

Global I-squared:
-------------------------

   i2.pair i2.cons
1 16.64527       0
```

```
-- Model fit (residual deviance):
    Dbar       pD      DIC
40.98702 31.98536 72.97238

37 data points, ratio 1.108, I^2 = 12%
```

3.2. Forest plots of the network meta-analysis for Absolute Neutrophil Count Recovery Time

Figure. S5. Forest plots of the network meta-analysis for absolute neutrophil count recovery time comparing different LA-G-CSFs with SA-G-CSF (rhG-CSF). The bars indicate mean difference (MD) and 95% credibility intervals (CrIs). LA-G-CSF = long-acting granulocyte colony-stimulating factor, SA-G-CSF = short-acting G-CSF.

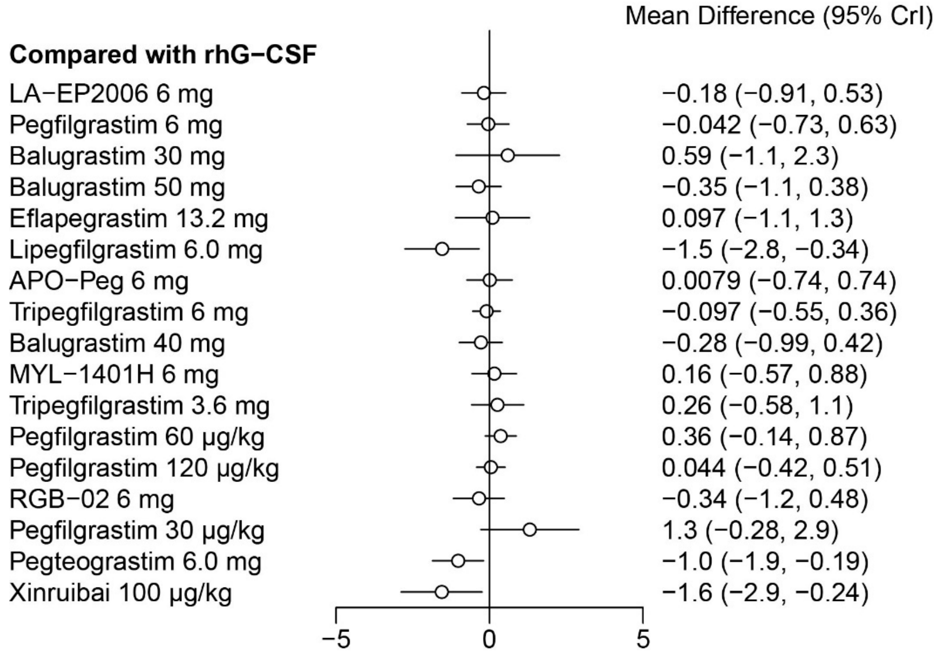

Figure. S6. Forest plots of the network meta-analysis for absolute neutrophil count recovery time comparing different LA-G-CSFs with ligpegfilgrasim 6 mg. The bars indicate mean difference (MD) and 95% credibility intervals (CrIs). LA-G-CSF = long-acting granulocyte colony-stimulating factor, SN = severe neutropenia.

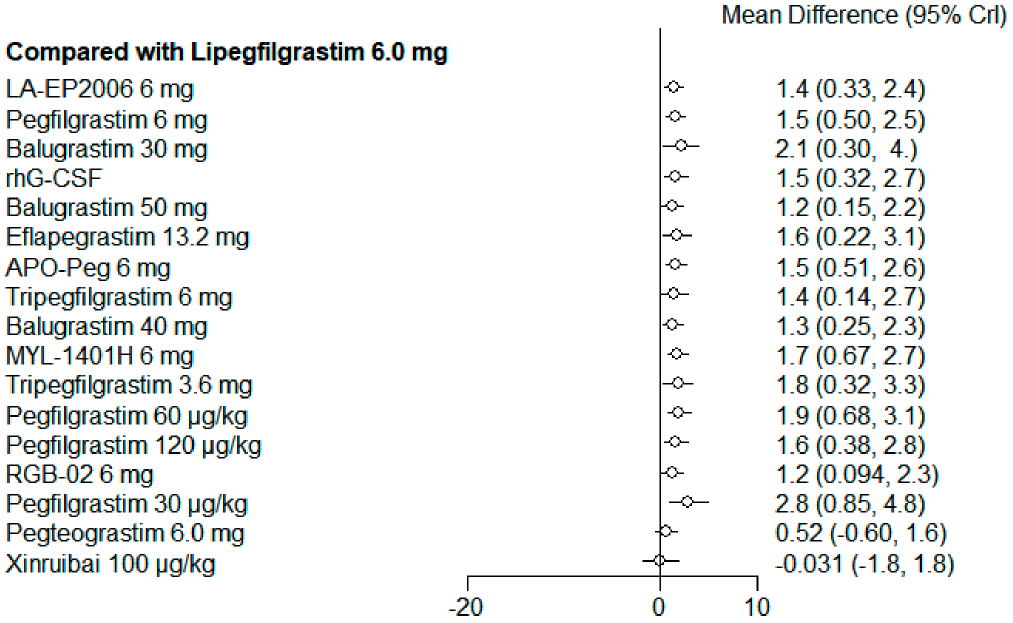
Mean Difference (95% CrI)
Compared with Lipegfilgrastim 6.0 mg
LA-EP2006 6 mg 1.4 (0.33, 2.4)
Pegfilgrastim 6 mg 1.5 (0.50, 2.5)
Balugrastim 30 mg 2.1 (0.30, 4.)
rhG-CSF 1.5 (0.32, 2.7)
Balugrastim 50 mg 1.2 (0.15, 2.2)
Eflapegrastim 13.2 mg 1.6 (0.22, 3.1)
APO-Peg 6 mg 1.5 (0.51, 2.6)
Tripegfilgrastim 6 mg 1.4 (0.14, 2.7)
Balugrastim 40 mg 1.3 (0.25, 2.3)
MYL-1401H 6 mg 1.7 (0.67, 2.7)
Tripegfilgrastim 3.6 mg 1.8 (0.32, 3.3)
Pegfilgrastim 60 µg/kg 1.9 (0.68, 3.1)
Pegfilgrastim 120 µg/kg 1.6 (0.38, 2.8)
RGB-02 6 mg 1.2 (0.094, 2.3)
Pegfilgrastim 30 µg/kg 2.8 (0.85, 4.8)
Pegteograstim 6.0 mg 0.52 (-0.60, 1.6)
Xinruibai 100 µg/kg -0.031 (-1.8, 1.8)
-20
0
10

3.3. Summary of confidence in network estimates

Figure. S7. CINeMA rankings comparing LA-G-CSFs with SA-G-CSF for absolute neutrophil count recovery time in drug-based analysis.

Comparison	Number of studies	Within-study bias	Reporting bias	Indirectness	Imprecision	Heterogeneity	Incoherence	Confidence rating
Pegfilgrastim 120 ug/kg:rhG-CSF	1	No concerns	Low risk	No concerns	No concerns	No concerns	No concerns	High
Pegfilgrastim 30 ug/kg:rhG-CSF	1	Major concerns	Low risk	No concerns	Some concerns	No concerns	No concerns	Low
Pegfilgrastim 6 mg:rhG-CSF	1	No concerns	Low risk	No concerns	No concerns	No concerns	No concerns	High
Pegfilgrastim 60 ug/kg:rhG-CSF	2	Some concerns	Low risk	No concerns	No concerns	No concerns	No concerns	High
rhG-CSF:Tripegfilgrastim 3.6 mg	1	Some concerns	Low risk	No concerns	No concerns	Some concerns	No concerns	Moderate
rhG-CSF:Tripegfilgrastim 6 mg	2	No concerns	Low risk	No concerns	No concerns	No concerns	No concerns	High
rhG-CSF:Xinruibai 100 ug/kg	1	No concerns	Low risk	No concerns	Some concerns	No concerns	No concerns	High
APO-Peg 6 mg:rhG-CSF	0	Some concerns	Low risk	No concerns	No concerns	No concerns	No concerns	High
Balugrastim 30 mg:rhG-CSF	0	No concerns	Low risk	No concerns	Some concerns	Some concerns	No concerns	Moderate
Balugrastim 40 mg:rhG-CSF	0	No concerns	Low risk	No concerns	No concerns	Some concerns	No concerns	High
Balugrastim 50 mg:rhG-CSF	0	No concerns	Low risk	No concerns	No concerns	Some concerns	No concerns	High
Eflapegrastim 13.2 mg:rhG-CSF	0	Some concerns	Low risk	No concerns	No concerns	No concerns	No concerns	High
LA-EP2006 6 mg:rhG-CSF	0	Some concerns	Low risk	No concerns	No concerns	Some concerns	No concerns	Moderate
Lipegfilgrastim 6 mg:rhG-CSF	0	No concerns	Low risk	No concerns	Some concerns	No concerns	No concerns	High
MYL-1401H 6 mg:rhG-CSF	0	No concerns	Low risk	No concerns	No concerns	Some concerns	No concerns	High
Pegteograstim 6 mg:rhG-CSF	0	No concerns	Low risk	No concerns	No concerns	Some concerns	No concerns	High
RGB-02 6 mg:rhG-CSF	0	Some concerns	Low risk	No concerns	No concerns	Some concerns	No concerns	Moderate

4. Incidence of Severe Neutropenia

4.1. Result of Heterogeneity and Consistency Analysis

```
> gelman.diag(SN1rslt)
Potential scale reduction factors:

        Point est. Upper C.I.
d.2.10           1          1
d.2.11           1          1
d.2.12           1          1
d.2.13           1          1
d.2.17           1          1
d.2.18           1          1
d.2.23           1          1
d.2.26           1          1
d.2.27           1          1
d.2.28           1          1
d.2.3            1          1
d.2.6            1          1
d.2.9            1          1
d.27.30          1          1
d.6.21           1          1
d.6.22           1          1
d.6.24           1          1
d.6.25           1          1
d.6.29           1          1
d.6.4            1          1
d.6.5            1          1
d.6.7            1          1
d.6.8            1          1

Multivariate psrf
1

-- Model fit (residual deviance):
     Dbar        pD       DIC
 65.70027  39.07942 104.77969

50 data points, ratio 1.314, I^2 = 25%
```

4.2. Forest plots of the network meta-analysis for Incidence of Severe Neutropenia

Figure. S8. Forest plots of the network meta-analysis for incidence of severe neutropenia comparing different LA-G-CSFs with SA-G-CSF (rhG-CSF). The bars indicate mean difference (MD) and 95% credibility intervals (CrIs). LA-G-CSF = long-acting granulocyte colony-stimulating factor, SA-G-CSF = short-acting G-CSF.

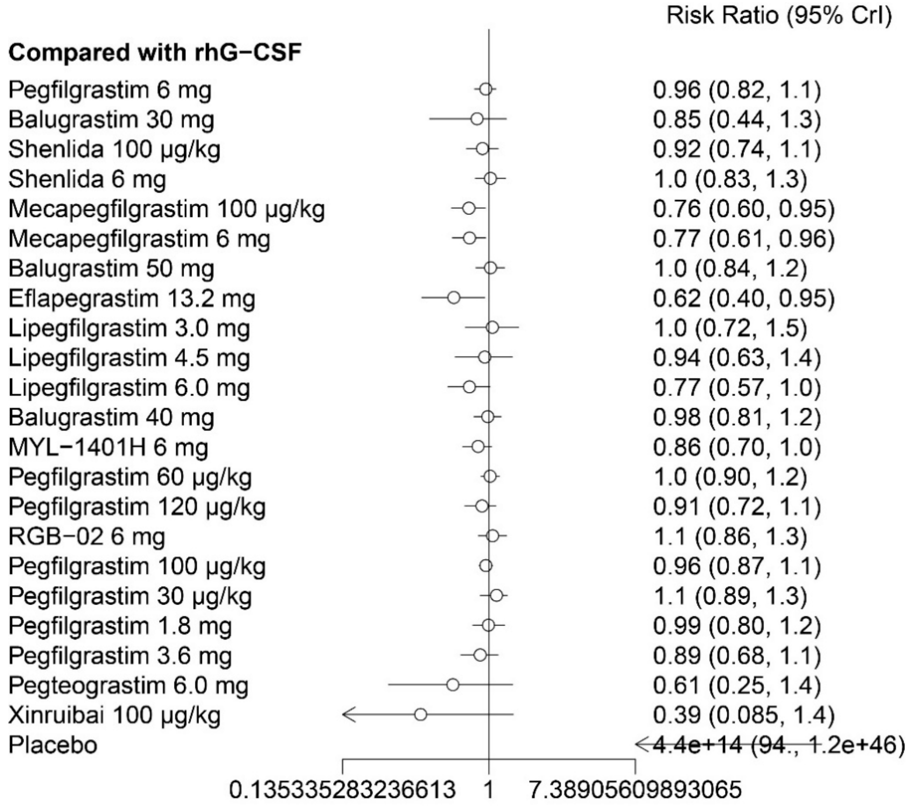

4.3. Summary of confidence in network estimates

Figure. S9. CINeMA rankings comparing LA-G-CSFs with SA-G-CSF for incidence of severe neutropenia in drug-based analysis.

Comparison	Number of studies	Within-study bias	Reporting bias	Indirectness	Imprecision	Heterogeneity	Incoherence	Confidence rating
Mecapegfilgrastim 100 ug/kg:rhG-CS	1	Some concerns	Low risk	No concers	Major concerns	No concers	No concers	Low
Mecapegfilgrastim 6 mg:rhG-CSF	1	Some concerns	Low risk	No concers	Major concerns	No concers	No concers	Low
Pegfilgrastim 100 ug/kg:rhG-CSF	3	Some concerns	Low risk	No concers	Major concerns	No concers	Major concerns	Very low
Pegfilgrastim 120 ug/kg:rhG-CSF	1	No concers	Low risk	No concers	Major concerns	No concers	No concers	Moderate
Pegfilgrastim 30 ug/kg:rhG-CSF	1	Major concerns	Low risk	No concers	Major concerns	No concers	No concers	Very low
Pegfilgrastim 6 mg:rhG-CSF	2	Some concerns	Low risk	No concers	Major concerns	No concers	No concers	Low
Pegfilgrastim 60 ug/kg:rhG-CSF	2	Some concerns	Low risk	No concers	Major concerns	No concers	No concers	Low
rhG-CSF:Shenlida 100 ug/kg	1	Some concerns	Low risk	No concers	Major concerns	No concers	No concers	Low
rhG-CSF:Shenlida 6 mg	1	Some concerns	Low risk	No concers	Major concerns	No concers	No concers	Low
rhG-CSF:Xinruibai 100 ug/kg	1	No concers	Low risk	No concers	Major concerns	No concers	No concers	Moderate
Balugrastim 30 mg:rhG-CSF	0	Some concerns	Low risk	No concers	Major concerns	No concers	No concers	Low
Balugrastim 40 mg:rhG-CSF	0	Some concerns	Low risk	No concers	Major concerns	No concers	No concers	Low
Balugrastim 50 mg:rhG-CSF	0	Some concerns	Low risk	No concers	Major concerns	No concers	No concers	Low
Eflapegrastim 13.2 mg:rhG-CSF	0	Major concerns	Low risk	No concers	Major concerns	No concers	No concers	Very low
Lipegfilgrastim 3.0 mg:rhG-CSF	0	No concers	Low risk	No concers	Major concerns	No concers	No concers	Moderate
Lipegfilgrastim 4.5 mg:rhG-CSF	0	No concers	Low risk	No concers	Major concerns	No concers	No concers	Moderate
Lipegfilgrastim 6.0 mg:rhG-CSF	0	No concers	Low risk	No concers	Major concerns	No concers	No concers	Moderate
MYL-1401H 6 mg:rhG-CSF	0	Some concerns	Low risk	No concers	Major concerns	No concers	No concers	Low
Pegfilgrastim 1.8 mg:rhG-CSF	0	No concers	Low risk	No concers	Major concerns	No concers	No concers	Moderate
Pegfilgrastim 3.6 mg:rhG-CSF	0	No concers	Low risk	No concers	Major concerns	No concers	No concers	Moderate
Pegteograstim 6.0 mg:rhG-CSF	0	Some concerns	Low risk	No concers	Major concerns	No concers	No concers	Low
Placebo:rhG-CSF	0	No concers	Low risk	No concers	No concers	No concers	No concers	High
RGB-02 6 mg:rhG-CSF	0	Major concerns	Low risk	No concers	Major concerns	No concers	No concers	Very low

5. Incidence of Febrile Neutropenia

5.1. Result of Heterogeneity and Consistency Analysis

```
> gelman.diag(FN1rslt)
Potential scale reduction factors:

       Point est. Upper C.I.
d.2.1           1       1.00
d.2.10          1       1.00
d.2.13          1       1.00
d.2.14          1       1.00
d.2.16          1       1.00
d.2.17          1       1.00
d.2.23          1       1.00
d.2.26          1       1.01
d.2.27          1       1.01
d.2.28          1       1.00
d.2.3           1       1.00
d.2.6           1       1.00
d.2.9           1       1.00
d.6.19          1       1.00
d.6.21          1       1.00
d.6.22          1       1.00
d.6.24          1       1.00
d.6.25          1       1.00
d.6.7           1       1.00
d.6.8           1       1.00

Multivariate psrf

1

Analysis of heterogeneity
=========================

Per-comparison I-squared:
-------------------------

   t1 t2  i2.pair  i2.cons  incons.p
1   1  2 2.422766 2.398215        NA
2  10  2       NA       NA        NA
3  13  2       NA       NA        NA
4  14 16       NA 0.000000 0.7396271
5  14  2       NA 0.000000 0.7840843
6  16 19       NA       NA        NA
7  16  2       NA 0.000000 0.7581924
8  16  6       NA 0.000000 0.7237453
9  17  2 0.000000 0.000000        NA
10 17  3       NA       NA        NA
11 17  9 0.000000 0.000000        NA
12 19  6       NA       NA        NA
13  2 23       NA       NA        NA
14  2 26       NA       NA        NA
15  2 27       NA       NA        NA
16  2 28       NA       NA        NA
17  2  3       NA       NA        NA
18  2  6 54.814052 24.060824 0.5241028
19  2  9 0.000000 0.000000        NA
20 21 22       NA       NA        NA
```

```
21 21 24  0.000000  0.000000 0.8369504
22 21 25        NA        NA        NA
23 21  6  0.000000  0.000000        NA
24 21  7        NA  0.000000 0.4902984
25 22 24        NA        NA        NA
26 22  6        NA        NA        NA
27 24 25        NA        NA        NA
28 24  6  0.000000  0.000000        NA
29 25  6        NA        NA        NA
30 26 27        NA        NA        NA
31  3  9        NA        NA        NA
32  6  7 25.694850 43.626485        NA
33  6  8        NA        NA        NA
34  7  8        NA        NA        NA

Global I-squared:
-------------------------

   i2.pair i2.cons
1 92.48992       0



-- Model fit (residual deviance):

    Dbar       pD      DIC
53.17457 41.17730 94.35188

55 data points, ratio 0.9668, I^2 = 0%


 Node-splitting analysis of inconsistency
 ========================================

    comparison  p.value  CrI
 1  d.2.6       0.523025
 2  -> direct            1.0 (0.48, 1.7)
 3  -> indirect          2.0 (-0.81, 5.7)
 4  -> network           1.1 (0.53, 1.7)
 5  d.2.14      0.751425
 6  -> direct            0.32 (-0.43, 1.1)
 7  -> indirect          -0.088 (-2.6, 2.3)
 8  -> network           0.28 (-0.44, 1.0)
 9  d.14.16     0.741575
 10 -> direct            0.86 (-0.87,  3.)
 11 -> indirect          0.45 (-1.2, 2.3)
 12 -> network           0.62 (-0.56, 1.9)
 13 d.2.16      0.745950
 14 -> direct            1.3 (-0.73, 4.6)
 15 -> indirect          0.83 (-0.64, 2.4)
 16 -> network           0.90 (-0.25, 2.2)
 17 d.6.16      0.525675
 18 -> direct            -0.83 (-4.2, 1.7)
 19 -> indirect          0.088 (-1.4, 1.8)
 20 -> network           -0.18 (-1.4, 1.2)
 21 d.7.21      0.027775
 22 -> direct              17. (0.15, 57.)
 23 -> indirect          -0.81 (-3.1, 1.1)
 24 -> network           -0.19 (-1.8, 1.4)
```

```
25 d.21.24     0.630675
26 -> direct            -0.75 (-1.8, 0.30)
27 -> indirect          -1.6 (-5.5, 2.2)
28 -> network           -0.72 (-1.6, 0.24)

>
```

5.2. Summary of confidence in network estimates

Figure. S10. CINeMA rankings comparing LA-G-CSFs with SA-G-CSF of incident FN in drug-based analysis.

Comparison	Number of studies	Within-study bias	Reporting bias	Indirectness	Imprecision	Heterogeneity	Incoherence	Confidence rating
Mecapegfilgrastim 100 ug/kg:rhG-CS[illegible]	2	Some concerns	Low risk	No concers	Major concerns	No concers	No concers	Low
Mecapegfilgrastim 6 mg:rhG-CSF	1	Some concerns	Low risk	No concers	Major concerns	No concers	No concers	Low
Pegfilgrastim 100 ug/kg:rhG-CSF	3	No concers	Low risk	No concers	Major concerns	No concers	No concers	Low
Pegfilgrastim 120 ug/kg:rhG-CSF	1	No concers	Low risk	No concers	Major concerns	No concers	No concers	Moderate
Pegfilgrastim 30 ug/kg:rhG-CSF	1	No concers	Low risk	No concers	Major concerns	No concers	No concers	Moderate
Pegfilgrastim 6 mg:rhG-CSF	2	Some concerns	Low risk	No concers	No concers	No concers	No concers	High
Pegfilgrastim 60 ug/kg:rhG-CSF	3	No concers	Low risk	No concers	Major concerns	No concers	No concers	Moderate
rhG-CSF:Tripegfilgrastim 3.6 mg	1	Some concerns	Low risk	No concers	Major concerns	No concers	No concers	Low
rhG-CSF:Tripegfilgrastim 6 mg	1	Some concerns	Low risk	No concers	Major concerns	No concers	No concers	Low
APO-Peg 6 mg:rhG-CSF	0	Some concerns	Low risk	No concers	Major concerns	No concers	No concers	Low
Balugrastim 30 mg:rhG-CSF	0	Some concerns	Low risk	No concers	Major concerns	No concers	No concers	Low
Balugrastim 40 mg:rhG-CSF	0	Some concerns	Low risk	No concers	Major concerns	No concers	No concers	Low
Balugrastim 50 mg:rhG-CSF	0	Some concerns	Low risk	No concers	Major concerns	No concers	No concers	Low
Eflapegrastim 13.2 mg:rhG-CSF	0	Some concerns	Low risk	No concers	No concers	No concers	No concers	High
LA-EP2006 6 mg:rhG-CSF	0	Some concerns	Low risk	No concers	No concers	No concers	No concers	High
Lipegfilgrastim 6 mg:rhG-CSF	0	Some concerns	Low risk	No concers	No concers	Major concerns	No concers	Low
Pegfilgrastim 1.8 mg:rhG-CSF	0	No concers	Low risk	No concers	Major concerns	No concers	No concers	Moderate
Pegfilgrastim 3.6 mg:rhG-CSF	0	No concers	Low risk	No concers	Major concerns	No concers	No concers	Moderate
Pegteograstim 6 mg:rhG-CSF	0	Some concerns	Low risk	No concers	Major concerns	No concers	No concers	Low
RGB-02 6 mg:rhG-CSF	0	Some concerns	Low risk	No concers	Major concerns	No concers	No concers	Low

6. Grade 3-4 Adverse Event

6.1. Result of Heterogeneity and Consistency Analysis

```
> gelman.diag(G34SErslt)
Potential scale reduction factors:

       Point est. Upper C.I.
d.2.1        1.00       1.00
d.2.13       1.00       1.00
d.2.17       1.00       1.00
d.2.18       1.03       1.08
d.2.23       1.00       1.00
d.2.28       1.00       1.00
d.2.3        1.00       1.00
d.2.9        1.00       1.00
d.5.10       1.00       1.00
d.6.16       1.00       1.00
d.6.2        1.00       1.00
d.6.24       1.00       1.00
d.6.31       1.00       1.00
d.6.32       1.00       1.00
d.6.33       1.00       1.00
d.6.4        1.00       1.01
d.6.5        1.00       1.00
d.6.7        1.00       1.00
d.6.8        1.00       1.00

Multivariate psrf

1.02
```

```
> summary(G34SEhe)
Analysis of heterogeneity
=========================

Per-comparison I-squared:
-------------------------

   t1 t2 i2.pair i2.cons incons.p
1   1  2      NA      NA       NA
2  10  5      NA      NA       NA
3  13  2      NA      NA       NA
4  16  6       0       0       NA
5  17  2       0       0       NA
6  17  3      NA      NA       NA
7  17  9       0       0       NA
8  18  2      NA      NA       NA
9   2 23      NA      NA       NA
10  2 28      NA      NA       NA
11  2  3      NA      NA       NA
12  2  6      NA      NA       NA
13  2  9       0       0       NA
14 24  6      NA      NA       NA
15  3  9      NA      NA       NA
16 31  6      NA      NA       NA
17 31  7      NA      NA       NA
18 32 33      NA      NA       NA
```

```
19 32   6        NA        NA         NA
20 33   6        NA        NA         NA
21  4   5        NA        NA         NA
22  4   6        NA        NA         NA
23  5   6        NA        NA         NA
24  6   7         0         0         NA
25  6   8        NA        NA         NA
26  7   8        NA        NA         NA

Global I-squared:
-------------------------

  i2.pair i2.cons
1       0       0


-- Model fit (residual deviance):

    Dbar       pD      DIC
36.88334 34.31702 71.20036

39 data points, ratio 0.9457, I^2 = 0%
```

6.2. Forest plots of the network meta-analysis for Grade 3-4 Adverse Event

Figure. S11(A). Forest plots of the network meta-analysis for incidence of Grade 3-4 Adverse Event comparing different LA-G-CSFs with SA-G-CSF (rhG-CSF). The bars indicate risk ratio (RR) and 95% credibility intervals (CrIs). LA-G-CSF = long-acting granulocyte colony-stimulating factor, SA-G-CSF = short-acting G-CSF.

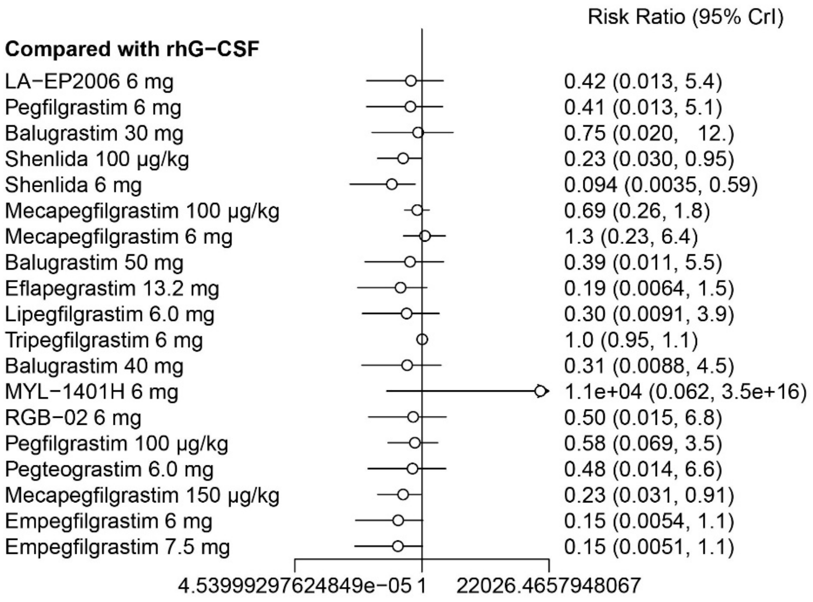

6.3. Summary of confidence in network estimates

Figure. S11(B). CINeMA rankings comparing LA-G-CSF with SA-G-CSF of G3-4 adverse event in drug-based analysis.

Comparison	Number of studies	Within-study bias	Reporting bias	Indirectness	Imprecision	Heterogeneity	Incoherence	Confidence rating
Empegfilgrastim 6 mg:rhG-CSF	1	No concerns	Low risk	No concerns	Major concerns	No concerns	No concerns	Moderate
Empegfilgrastim 7.5 mg:rhG-CSF	1	No concerns	Low risk	No concerns	Major concerns	No concerns	No concerns	Moderate
Mecapegfilgrastim 100 ug/kg:rhG-CSF	2	Some concerns	Low risk	No concerns	Major concerns	No concerns	No concerns	Low
Mecapegfilgrastim 150 ug/kg:rhG-CSF	1	Some concerns	Low risk	No concerns	Major concerns	No concerns	No concerns	Low
Mecapegfilgrastim 6 mg:rhG-CSF	1	Some concerns	Low risk	No concerns	Major concerns	No concerns	No concerns	Low
Pegfilgrastim 100 ug/kg:rhG-CSF	1	No concerns	Low risk	No concerns	Major concerns	No concerns	No concerns	Moderate
Pegfilgrastim 6 mg:rhG-CSF	1	Some concerns	Low risk	No concerns	Major concerns	No concerns	No concerns	Low
rhG-CSF:Shenlida 100 ug/kg	1	Some concerns	Low risk	No concerns	Major concerns	No concerns	No concerns	Low
rhG-CSF:Shenlida 6 mg	1	Some concerns	Low risk	No concerns	Major concerns	No concerns	No concerns	Low
rhG-CSF:Tripegfilgrastim 6 mg	1	No concerns	Low risk	No concerns	Major concerns	No concerns	No concerns	Moderate
Balugrastim 30 mg:rhG-CSF	0	Some concerns	Low risk	No concerns	Major concerns	No concerns	No concerns	Low
Balugrastim 40 mg:rhG-CSF	0	Some concerns	Low risk	No concerns	Major concerns	No concerns	No concerns	Low
Balugrastim 50 mg:rhG-CSF	0	Some concerns	Low risk	No concerns	Major concerns	No concerns	No concerns	Low
Eflapegrastim 13.2 mg:rhG-CSF	0	Some concerns	Low risk	No concerns	Major concerns	No concerns	No concerns	Low
LA-EP2006 6 mg:rhG-CSF	0	Some concerns	Low risk	No concerns	Major concerns	No concerns	No concerns	Low
Lipegfilgrastim 6.0 mg:rhG-CSF	0	Some concerns	Low risk	No concerns	Major concerns	No concerns	No concerns	Low
MYL-1401H 6 mg:rhG-CSF	0	Some concerns	Low risk	No concerns	Major concerns	No concerns	No concerns	Low
Pegteograstim 6.0 mg:rhG-CSF	0	Some concerns	Low risk	No concerns	Major concerns	No concerns	No concerns	Low
RGB-02 6 mg:rhG-CSF	0	Major concerns	Low risk	No concerns	Major concerns	No concerns	No concerns	Very low

7. SUCRA results (percentages) for other drugs of pegfilgrastim and its biosimilar.

Supplementary Table S1. SUCRA results (percentages) for other drugs of LA-G-CSFs.

Drug	Outcome				
	Duration of SN	incidence of SN	incidence of FN	ANC recovery time	Grade 3-4 AE
Tripegfilgrastim 6 mg	52.3%	-	34.7%	51.3%	23.7%
Tripegfilgrastim 3.6 mg	58.6%	-	15.3%	30.6%	-
MYL-1401H 6 mg	42.2%	66.4%	-	27.5%	3.8%
APO-Peg 6 mg	30.4%	-	57.2%	39.2%	-
Pegfilgrastim 1.8 mg	4.7%	36.1%	39.6%	-	-
Pegfilgrastim 3.6 mg	36.0%	6-	27.4%	-	-
Pegfilgrastim 30 μg/kg	4.0%	17.9%	51.5%	7.0%	-
Pegfilgrastim 60 μg/kg	28.4%	31.0%	20.6%	19.8%	-
Lipegfilgrastim 3 mg	22.4%	29.9%	-	-	-
Lipegfilgrastim 4.5 mg	45.1%	46.7%	-	-	-
Mecapegfilgrastim 150 μg/kg	-	-	-	-	66.7%

SUCRA: surface under the cumulative ranking curve. SUCRA values can range from 0% (i.e., the treatment always ranks last) to 100% (i.e., the treatment always ranks first). ANC = absolute neutrophil count, FN = febrile neutropenia, SN = severe neutropenia, AE = adverse event.

Dose-based Analysis

8. Duration of Severe Neutropenia

8.1. Result of Heterogeneity and Consistency Analysis

```
> gelman.diag(DSNrslt)
Potential scale reduction factors:

        Point est. Upper C.I.
d.1.11           1          1
d.1.13           1          1
d.1.16           1          1
d.1.6            1          1
d.1.7            1          1
d.1.8            1          1
d.13.10          1          1
d.13.12          1          1
d.13.14          1          1
d.13.15          1          1
d.13.3           1          1
d.13.4           1          1
d.13.9           1          1

Multivariate psrf

1

Analysis of heterogeneity
=========================

Per-comparison I-squared:
-------------------------

   t1 t2 i2.pair  i2.cons     incons.p
1   1 11       NA  0.00000 5.457391e-01
2   1 13 97.83748 97.85682           NA
3   1 16       NA       NA           NA
4   1  6  0.00000  0.00000           NA
5   1  7 44.35020 56.28575           NA
6   1  8       NA       NA           NA
7  10 12       NA       NA           NA
8  10 13       NA       NA           NA
9  11 13  0.00000  0.00000           NA
10 11  9       NA       NA           NA
11 12 13       NA       NA           NA
12 13 14  0.00000  0.00000           NA
13 13 15  0.00000  0.00000           NA
14 13 16       NA       NA           NA
15 13  3  0.00000  0.00000           NA
16 13  4  0.00000  0.00000           NA
17 13  7  0.00000 86.68589 9.940508e-05
18 13  9       NA       NA           NA
19 15  3       NA  0.00000 8.153310e-01
20 15  4       NA  0.00000 7.398308e-01
21  3  4 25.52038 31.69914           NA
22  6  7  0.00000  0.00000           NA
23  6  8       NA       NA           NA
```

```
24   7   8         NA        NA            NA

Global I-squared:
-------------------------

   i2.pair  i2.cons
1 93.72622 90.88862

    Dbar        pD       DIC
380.18057  32.07249 412.25306

50 data points, ratio 7.604, I^2 = 87%

> summary(DSNns)
Node-splitting analysis of inconsistency
========================================

   comparison  p.value  CrI
1  d.1.11      0.478625
2  -> direct            -0.28 (-1.1, 0.50)
3  -> indirect          -0.67 (-1.4, 0.084)
4  -> network           -0.54 (-1.1, -0.023)
5  d.7.13      0.000075
6  -> direct            0.22 (-0.031, 0.47)
7  -> indirect          -0.75 (-1.0, -0.49)
8  -> network           -0.26 (-0.44, -0.081)
9  d.3.15      0.674375
10 -> direct            -0.51 (-2.0, 1.0)
11 -> indirect          -0.17 (-0.64, 0.31)
12 -> network           -0.18 (-0.63, 0.28)
13 d.4.15      0.550225
14 -> direct            0.10 (-1.4, 1.6)
15 -> indirect          -0.39 (-0.96, 0.19)
16 -> network           -0.30 (-0.83, 0.23)
```

8.2. Forest plots of the network meta-analysis for Duration of Severe Neutropenia

Figure. S12. Forest plots of the network meta-analysis for duration of severe neutropenia comparing different doses of LA-G-CSFs (PEG-rhG-CSF) with 6 mg. The bars indicate mean difference (MD) and 95% credibility intervals (CrIs). LA-G-CSF = long-acting granulocyte colony-stimulating factor.

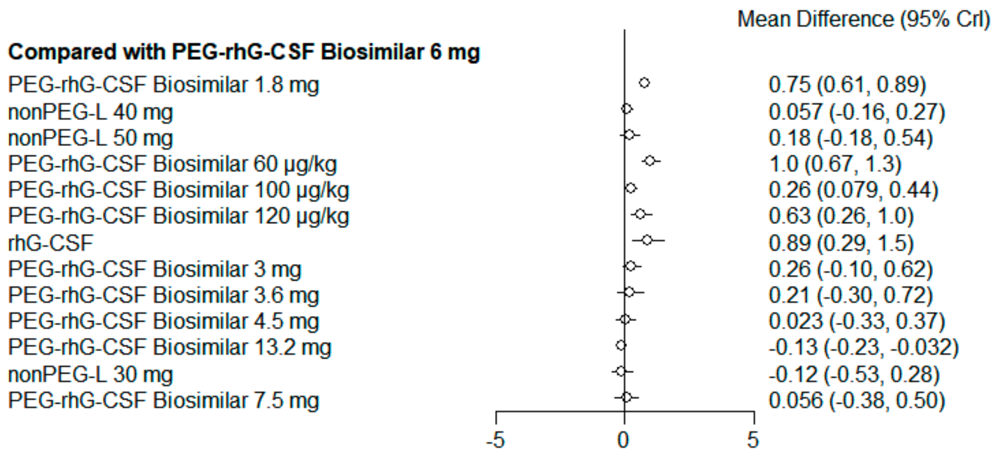

8.3. Summary of confidence in network estimates

Figure. S13. CINeMA rankings comparing LA-G-CSF (PEG-rhG-CSF) 6 mg with other doses for duration of SN in dose-based analysis.

Comparison	Number of studies	Within-study bias	Reporting bias	Indirectness	Imprecision	Heterogeneity	Incoherence	Confidence rating
PEG-rhG-CSF Biosimilar 1.8 mg:PEG-rhG-CSF Biosimilar 6 mg	1	No concerns	Low risk	No concerns	Some concerns	No concerns	No concerns	High
PEG-rhG-CSF Biosimilar 100 ug/kg:PEG-rhG-CSF Biosimilar 6 mg	2	Some concerns	Low risk	No concerns	No concerns	No concerns	No concerns	High
PEG-rhG-CSF Biosimilar 13.2 mg:PEG-rhG-CSF Biosimilar 6 mg	1	Some concerns	Low risk	No concerns	No concerns	Major concerns	No concerns	Low
PEG-rhG-CSF Biosimilar 3 mg:PEG-rhG-CSF Biosimilar 6 mg	1	No concerns	Low risk	No concerns	Some concerns	No concerns	No concerns	High
PEG-rhG-CSF Biosimilar 3.6 mg:PEG-rhG-CSF Biosimilar 6 mg	3	Some concerns	Low risk	No concerns	No concerns	No concerns	No concerns	High
PEG-rhG-CSF Biosimilar 4.5 mg:PEG-rhG-CSF Biosimilar 6 mg	1	No concerns	Low risk	No concerns	No concerns	Major concerns	No concerns	Moderate
PEG-rhG-CSF Biosimilar 6 mg:PEG-rhG-CSF Biosimilar 7.5 mg	1	No concerns	Low risk	No concerns	No concerns	Some concerns	No concerns	High
nonPEG-L 30 mg:PEG-rhG-CSF Biosimilar 6 mg	2	No concerns	Low risk	No concerns	No concerns	Some concerns	No concerns	High
nonPEG-L 40 mg:PEG-rhG-CSF Biosimilar 6 mg	3	No concerns	Low risk	No concerns	No concerns	No concerns	No concerns	High
nonPEG-L 50 mg:PEG-rhG-CSF Biosimilar 6 mg	2	Some concerns	Low risk	No concerns	No concerns	Some concerns	No concerns	Moderate
PEG-rhG-CSF Biosimilar 6 mg:rhG-CSF	8	Some concerns	Low risk	No concerns	No concerns	Some concerns	No concerns	Moderate
PEG-rhG-CSF Biosimilar 120 ug/kg:PEG-rhG-CSF Biosimilar 6 mg	0	No concerns	Low risk	No concerns	No concerns	Some concerns	No concerns	High
PEG-rhG-CSF Biosimilar 60 ug/kg:PEG-rhG-CSF Biosimilar 6 mg	0	Some concerns	Low risk	No concerns	Some concerns	No concerns	No concerns	Moderate

9. Absolute Neutrophil Count Recovery Time

9.1. Result of Heterogeneity and Consistency Analysis

```
> gelman.diag(ANCrslt)
Potential scale reduction factors:

         Point est. Upper C.I.
d.1.11            1          1
d.1.13            1          1
d.1.6             1          1
d.1.7             1          1
d.1.8             1          1
d.13.10           1          1
d.13.12           1          1
d.13.14           1          1
d.13.15           1          1
d.13.3            1          1
d.13.4            1          1

Multivariate psrf

1

>
```

```
Analysis of heterogeneity
=========================

Per-comparison I-squared:
-------------------------

   t1 t2  i2.pair  i2.cons  incons.p
1   1 11       NA       NA        NA
2   1 13  0.00000  0.00000        NA
3   1  6       NA  0.00000 0.6756348
4   1  7 62.37488 65.01769        NA
5   1  8       NA       NA        NA
6  10 12       NA       NA        NA
7  10 13       NA       NA        NA
8  11 13       NA       NA        NA
9  12 13       NA       NA        NA
10 13 14       NA       NA        NA
11 13 15       NA       NA        NA
12 13  3 61.05948 61.61968        NA
13 13  4  0.00000  0.00000        NA
14 15  3       NA       NA        NA
15 15  4       NA       NA        NA
16  3  4 21.79766 55.52927        NA
17  6  7  0.00000  0.00000        NA
18  6  8       NA       NA        NA
19  7  8       NA       NA        NA

Global I-squared:
-------------------------
```

```
  i2.pair i2.cons
1 27.8861       0

Model fit (residual deviance):

    Dbar       pD      DIC 
32.88092 20.97660 53.85752 

27 data points, ratio 1.218, I^2 = 21%

> summary(ANCns)
Node-splitting analysis of inconsistency
========================================

  comparison  p.value CrI              
1 d.1.6       0.30625                  
2 -> direct           0.23 (-0.35, 0.80)
3 -> indirect         -0.78 (-2.6, 1.1)
4 -> network          0.15 (-0.39, 0.70)
```

9.2. Forest plots of the network meta-analysis for Absolute Neutrophil Count Recovery Time

Figure. S14. Forest plots of the network meta-analysis for absolute neutrophil count recovery time comparing different doses of LA-G-CSFs (PEG-rhG-CSF) with 6 mg. The bars indicate mean difference (MD) and 95% credibility intervals (CrIs). LA-G-CSF = long-acting granulocyte colony-stimulating factor, nonPEG-L = non polyethylene glycol LA-G-CSF

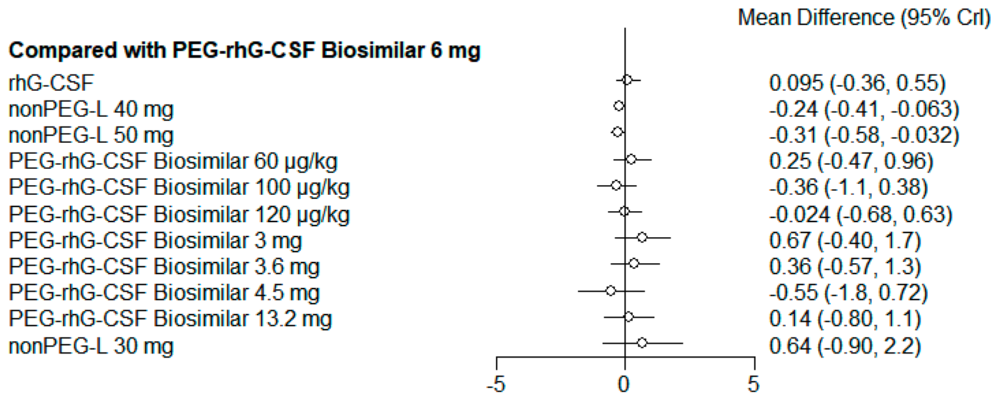

9.3. Summary of confidence in network estimates

Figure. S15. CINeMA rankings comparing LA-G-CSF (PEG-rhG-CSF) 6 mg with other doses for absolute neutrophil count recovery time in dose-based analysis.

Comparison	Number of studies	Within-study bias	Reporting bias	Indirectness	Imprecision	Heterogeneity	Incoherence	Confidence rating
PEG-rhG-CSF Biosimilar 13.2 mg:PEG-rhG-CSF Biosimilar 6 mg	1	Some concerns	Low risk	No concerns	No concerns	No concerns	No concerns	High
PEG-rhG-CSF Biosimilar 3 mg:PEG-rhG-CSF Biosimilar 6 mg	1	No concerns	Low risk	No concerns	No concerns	No concerns	No concerns	High
PEG-rhG-CSF Biosimilar 3.6 mg:PEG-rhG-CSF Biosimilar 6 mg	1	Some concerns	Low risk	No concerns	No concerns	Some concerns	No concerns	High
PEG-rhG-CSF Biosimilar 4.5 mg:PEG-rhG-CSF Biosimilar 6 mg	1	No concerns	Low risk	No concerns	No concerns	No concerns	No concerns	High
nonPEG-L 30 mg:PEG-rhG-CSF Biosimilar 6 mg	1	No concerns	Low risk	No concerns	No concerns	Some concerns	No concerns	High
nonPEG-L 40 mg:PEG-rhG-CSF Biosimilar 6 mg	3	No concerns	Low risk	No concerns	No concerns	No concerns	No concerns	High
nonPEG-L 50 mg:PEG-rhG-CSF Biosimilar 6 mg	2	Some concerns	Low risk	No concerns	No concerns	No concerns	No concerns	High
PEG-rhG-CSF Biosimilar 6 mg:rhG-CSF?	2	No concerns	Low risk	No concerns	No concerns	No concerns	No concerns	High
PEG-rhG-CSF Biosimilar 100 ug/kg:PEG-rhG-CSF Biosimilar 6 mg	0	No concerns	Low risk	No concerns	No concerns	Some concerns	No concerns	High
PEG-rhG-CSF Biosimilar 120 ug/kg:PEG-rhG-CSF Biosimilar 6 mg	0	No concerns	Low risk	No concerns	No concerns	No concerns	No concerns	High
PEG-rhG-CSF Biosimilar 60 ug/kg:PEG-rhG-CSF Biosimilar 6 mg	0	No concerns	Low risk	No concerns	No concerns	No concerns	No concerns	High

10. Incidence of Severe Neutropenia

10.1. Result of Heterogeneity and Consistency Analysis

```
> gelman.diag(SN1rslt)
Potential scale reduction factors:

        Point est. Upper C.I.
d.1.13           1          1
d.1.5            1          1
d.1.6            1          1
d.1.7            1          1
d.1.8            1          1
d.13.10          1          1
d.13.11          1          1
d.13.12          1          1
d.13.14          1          1
d.13.15          1          1
d.13.3           1          1
d.13.4           1          1
d.13.9           1          1

Multivariate psrf

1

Analysis of heterogeneity
=========================

Per-comparison I-squared:
-------------------------

   t1 t2  i2.pair  i2.cons  incons.p
1   1 13 46.26784 46.54321        NA
2   1  5       NA       NA        NA
3   1  6 58.71394 58.73887        NA
4   1  7  0.00000  0.00000        NA
5   1  8       NA       NA        NA
6  10 12       NA       NA        NA
7  10 13       NA       NA        NA
8  11 13       NA       NA        NA
9  11  9       NA       NA        NA
10 12 13       NA       NA        NA
11 13 14       NA       NA        NA
12 13 15       NA       NA        NA
13 13  3  0.00000  0.00000        NA
14 13  4  0.00000  0.00000        NA
15 13  7  0.00000  0.00000 0.7990651
16 13  9       NA       NA        NA
17 15  3       NA       NA        NA
18 15  4       NA       NA        NA
19  3  4  0.00000  0.00000        NA
20  5  6       NA       NA        NA
21  5  7       NA       NA        NA
22  6  7 49.06497 51.17972        NA
23  6  8       NA       NA        NA
24  7  8       NA       NA        NA

Global I-squared:
```

```
-------------------------

   i2.pair i2.cons
1 3.303477       0

-- Model fit (residual deviance):

    Dbar       pD      DIC
43.00508 24.16065 67.16573

37 data points, ratio 1.162, I^2 = 16%


 Node-splitting analysis of inconsistency
 ========================================

   comparison  p.value CrI
 1 d.7.13      0.9874
 2 -> direct           0.067 (-0.098, 0.23)
 3 -> indirect         0.069 (-0.10, 0.25)
 4 -> network          0.050 (-0.065, 0.17)

 >
```

10.2. Forest plots of the network meta-analysis for Incidence of Severe Neutropenia

Figure. S16. Forest plots of the network meta-analysis for incidence of severe neutropenia comparing different doses of LA-G-CSFs (PEG-rhG-CSF) with 6 mg. The bars indicate Risk Ratio (RR) and 95% credibility intervals (CrIs). LA-G-CSF = long-acting granulocyte colony-stimulating factor, nonPEG-L = non polyethylene glycol LA-G-CSF

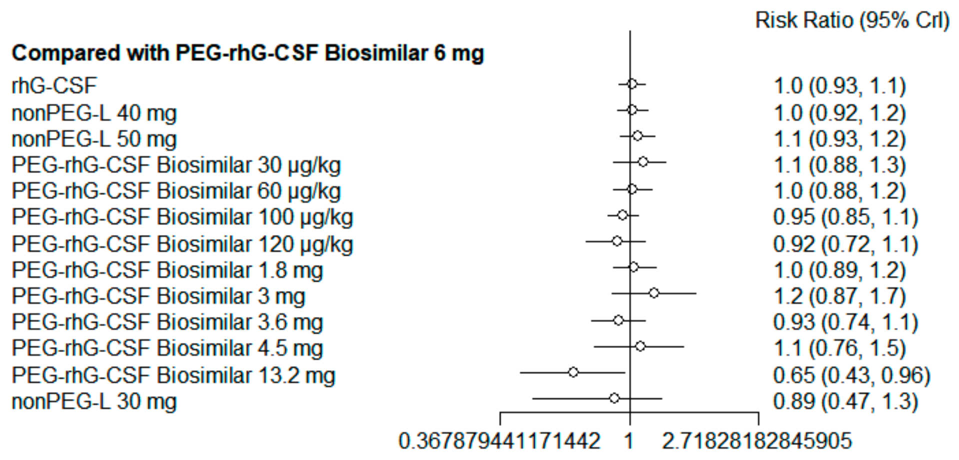

10.3. Summary of confidence in network estimates

Figure. S17. CINeMA rankings comparing LA-G-CSF (PEG-rhG-CSF) 6 mg with other doses for incidence of severe neutropenia in dose-based analysis.

Comparison	Number of studies	Within-study bias	Reporting bias	Indirectness	Imprecision	Heterogeneity	Incoherence	Confidence rating
PEG-rhG-CSF Biosimilar 1.8 mg:PEG-rhG-CSF Biosimilar 6 mg	1	No concerns	Low risk	No concerns	Major concerns	No concerns	Major concerns	Low
PEG-rhG-CSF Biosimilar 3 mg:PEG-rhG-CSF Biosimilar 6 mg	1	No concerns	Low risk	No concerns	Major concerns	No concerns	Major concerns	Low
PEG-rhG-CSF Biosimilar 4.5 mg:PEG-rhG-CSF Biosimilar 6 mg	1	No concerns	Low risk	No concerns	Major concerns	No concerns	Major concerns	Low
PEG-rhG-CSF Biosimilar 6 mg:rhG-CSF	3	Some concerns	Low risk	No concerns	No concerns	Major concerns	Major concerns	Low
PEG-rhG-CSF Biosimilar 120 ug/kg:PEG-rhG-CSF Biosimilar 6 mg	0	Some concerns	Low risk	No concerns	Major concerns	No concerns	Major concerns	Low
PEG-rhG-CSF Biosimilar 30 ug/kg:PEG-rhG-CSF Biosimilar 6 mg	0	Some concerns	Low risk	No concerns	Major concerns	No concerns	Major concerns	Low
PEG-rhG-CSF Biosimilar 60 ug/kg:PEG-rhG-CSF Biosimilar 6 mg	0	Some concerns	Low risk	No concerns	Major concerns	No concerns	Major concerns	Low
PEG-rhG-CSF Biosimilar 100 ug/kg:PEG-rhG-CSF Biosimilar 6 mg	2	Some concerns	Low risk	No concerns	Major concerns	No concerns	No concerns	Moderate
PEG-rhG-CSF Biosimilar 3.6 mg:PEG-rhG-CSF Biosimilar 6 mg	1	No concerns	Low risk	No concerns	No concerns	No concerns	Major concerns	Moderate
nonPEG-L 30 mg:PEG-rhG-CSF Biosimilar 6 mg	1	No concerns	Low risk	No concerns	Major concerns	No concerns	No concerns	Moderate
nonPEG-L 40 mg:PEG-rhG-CSF Biosimilar 6 mg	3	No concerns	Low risk	No concerns	Major concerns	No concerns	No concerns	Moderate
nonPEG-L 50 mg:PEG-rhG-CSF Biosimilar 6 mg	2	Some concerns	Low risk	No concerns	Major concerns	No concerns	No concerns	Moderate
PEG-rhG-CSF Biosimilar 13.2 mg:PEG-rhG-CSF Biosimilar 6 mg	1	Major concerns	Low risk	No concerns	Major concerns	No concerns	Major concerns	Very low

11. Incidence of Febrile Neutropenia

11.1. Result of Heterogeneity and Consistency Analysis

```
> gelman.diag(FN1rslt)
Potential scale reduction factors:

        Point est. Upper C.I.
d.1.11           1          1
d.1.13           1          1
d.1.17           1          1
d.1.5            1          1
d.1.6            1          1
d.1.7            1          1
d.1.8            1          1
d.13.14          1          1
d.13.15          1          1
d.13.3           1          1
d.13.4           1          1
d.13.9           1          1

Multivariate psrf

1

-- Model fit (residual deviance):

   Dbar      pD     DIC
37.5995 25.2152 62.8147

35 data points, ratio 1.074, I^2 = 10%


Analysis of heterogeneity
=========================

Per-comparison I-squared:
-------------------------

   t1 t2  i2.pair   i2.cons  incons.p
1   1 11       NA  0.000000 0.8670280
2   1 13  0.00000  0.000000        NA
3   1 17       NA        NA        NA
4   1  5       NA        NA        NA
5   1  6 13.95825 27.902936        NA
6   1  7 15.69391 16.225733        NA
7   1  8       NA        NA        NA
8  11 13  0.00000  0.000000        NA
9  11  9       NA        NA        NA
10 13 14       NA        NA        NA
11 13 15       NA        NA        NA
12 13  3  0.00000  0.000000        NA
13 13  4  0.00000  0.000000        NA
14 13  7       NA  4.501285 0.3055117
15 13  9       NA        NA        NA
16 15  3       NA        NA        NA
17 15  4       NA        NA        NA
18 17  7       NA        NA        NA
```

```
19   3   4   0.00000   0.000000         NA
20   5   6        NA         NA         NA
21   5   7        NA         NA         NA
22   6   7   0.00000   0.000000         NA
23   6   8        NA         NA         NA
24   7   8        NA         NA         NA

Global I-squared:
-------------------------

  i2.pair i2.cons
1       0       0


Node-splitting analysis of inconsistency
========================================

  comparison  p.value  CrI
1 d.1.11      0.994350
2 -> direct            0.53 (-1.2, 2.6)
3 -> indirect          0.49 (-1.9, 3.8)
4 -> network           0.40 (-0.95, 1.8)
5 d.7.13      0.001175
6 -> direct             -26. (-81., -2.9)
7 -> indirect          -0.032 (-1.1, 0.98)
8 -> network           -0.54 (-1.5, 0.37)
```

11.2. Forest plots of the network meta-analysis for Incidence of Febrile Neutropenia

Figure. S18. Forest plots of the network meta-analysis for incidence of febrile neutropenia comparing different doses of LA-G-CSFs (PEG-rhG-CSF) with 6 mg. The bars indicate Risk Ratio (RR) and 95% credibility intervals (CrIs). LA-G-CSF = long-acting granulocyte colony-stimulating factor, nonPEG-L = non polyethylene glycol LA-G-CSF

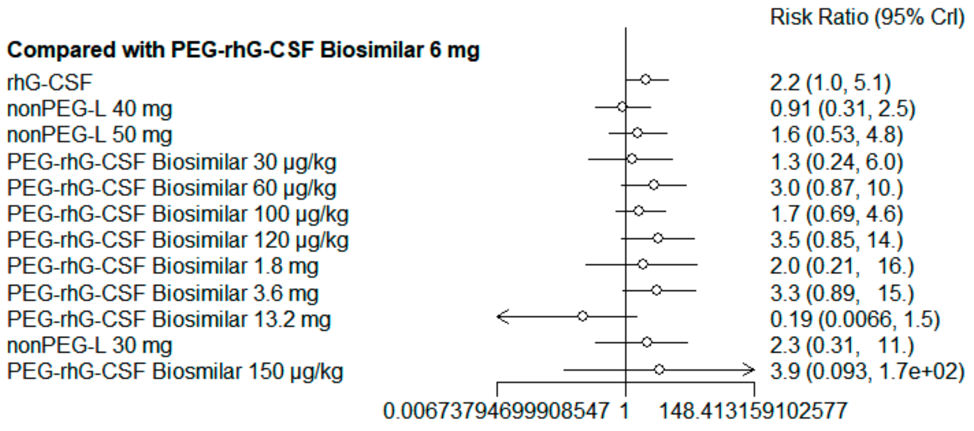

11.3. Summary of confidence in network estimates

Figure. S19. CINeMA rankings comparing LA-G-CSF (PEG-rhG-CSF) 6 mg with other doses for incidence of febrile neutropenia in dose-based analysis.

Comparison	Number of studies	Within-study bias	Reporting bias	Indirectness	Imprecision	Heterogeneity	Incoherence	Confidence rating
PEG-rhG-CSF Biosimilar 1.8 mg:PEG-rhG-CSF Biosimilar 6 mg	1	No concerns	Low risk	No concerns	Major concerns	No concerns	No concerns	Moderate
PEG-rhG-CSF Biosimilar 100 ug/kg:PEG-rhG-CSF Biosimilar 6 mg	1	Some concerns	Low risk	No concerns	Major concerns	No concerns	No concerns	Low
PEG-rhG-CSF Biosimilar 13.2 mg:PEG-rhG-CSF Biosimilar 6 mg	1	Some concerns	Low risk	No concerns	Major concerns	No concerns	No concerns	Low
PEG-rhG-CSF Biosimilar 3.6 mg:PEG-rhG-CSF Biosimilar 6 mg	2	Some concerns	Low risk	No concerns	Major concerns	No concerns	No concerns	Low
nonPEG-L 30 mg:PEG-rhG-CSF Biosimilar 6 mg	1	No concerns	Low risk	No concerns	Major concerns	No concerns	No concerns	Moderate
nonPEG-L 40 mg:PEG-rhG-CSF Biosimilar 6 mg	3	No concerns	Low risk	No concerns	Major concerns	No concerns	No concerns	Moderate
nonPEG-L 50 mg:PEG-rhG-CSF Biosimilar 6 mg	2	Some concerns	Low risk	No concerns	Major concerns	No concerns	No concerns	Low
PEG-rhG-CSF Biosimilar 6 mg:rhG-CSF	3	Some concerns	Low risk	No concerns	Major concerns	No concerns	No concerns	Low
PEG-rhG-CSF Biosimilar 120 ug/kg:PEG-rhG-CSF Biosimilar 6 mg	0	Some concerns	Low risk	No concerns	Major concerns	No concerns	No concerns	Low
PEG-rhG-CSF Biosimilar 30 ug/kg:PEG-rhG-CSF Biosimilar 6 mg	0	Some concerns	Low risk	No concerns	Major concerns	No concerns	No concerns	Low
PEG-rhG-CSF Biosimilar 60 ug/kg:PEG-rhG-CSF Biosimilar 6 mg	0	Some concerns	Low risk	No concerns	Major concerns	No concerns	No concerns	Low
PEG-rhG-CSF Biosimilar 6 mg:PEG-rhG-CSF Biosmilar 150 ug/kg	0	Some concerns	Low risk	No concerns	Major concerns	No concerns	No concerns	Low

12. Grade 3-4 Adverse Event

12.1. Result of Heterogeneity and Consistency Analysis

```
> gelman.diag(G34SErslt)
Potential scale reduction factors:

        Point est. Upper C.I.
d.13.1           1          1
d.13.14          1          1
d.13.15          1          1
d.13.16          1          1
d.13.3           1          1
d.13.4           1          1
d.13.7           1          1

Multivariate psrf

1

Analysis of heterogeneity
=========================

Per-comparison I-squared:
-------------------------

   t1 t2  i2.pair i2.cons  incons.p
1   1 13 32.09957 32.2696        NA
2   1 16       NA      NA        NA
3   1  7  0.00000  0.0000        NA
4  13 14       NA      NA        NA
5  13 15       NA      NA        NA
6  13 16       NA      NA        NA
7  13  3  0.00000  0.0000        NA
8  13  4  0.00000  0.0000        NA
9  13  7       NA  0.0000 0.5827079
10 15  3       NA      NA        NA
11 15  4       NA      NA        NA
12  3  4  0.00000  0.0000        NA

Global I-squared:
-------------------------

  i2.pair i2.cons
1       0       0



-- Model fit (residual deviance):

    Dbar       pD      DIC
27.48058 15.38905 42.86963

23 data points, ratio 1.195, I^2 = 20%



Node-splitting analysis of inconsistency
========================================
```

```
  comparison  p.value CrI
1 d.7.13      0.35485
2 -> direct           -0.90 (-4.4, 1.6)
3 -> indirect         0.57 (-1.2, 2.6)
4 -> network          0.72 (-0.34, 2.0)
```

12.2. Forest plots of the network meta-analysis for Grade 3-4 Adverse Event

Figure. S20. Forest plots of the network meta-analysis for grade 3-4 adverse event comparing different doses of LA-G-CSFs (PEG-rhG-CSF Biosimilar) with 6 mg. The bars indicate Risk Ratio (RR) and 95% credibility intervals (CrIs). LA-G-CSF = long-acting granulocyte colony-stimulating factor, nonPEG-L = non polyethylene glycol LA-G-CSF

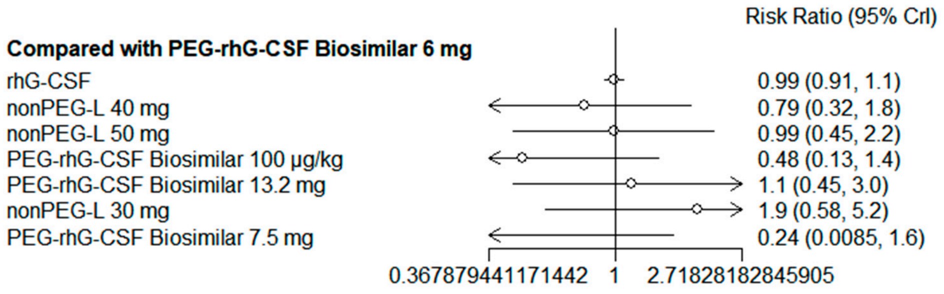

Figure. S21. Forest plots of the network meta-analysis for grade 3-4 adverse event comparing different doses of LA-G-CSFs (PEG-rhG-CSF Biosimilar) with 13.2 mg. The bars indicate Risk Ratio (RR) and 95% credibility intervals (CrIs). LA-G-CSF = long-acting granulocyte colony-stimulating factor, nonPEG-L = non polyethylene glycol LA-G-CSF

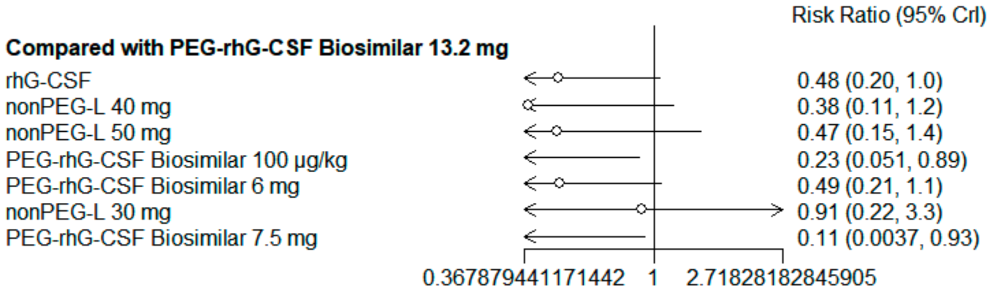

12.3. Summary of confidence in network estimates

Figure. S22. CINeMA rankings comparing LA-G-CSF (PEG-rhG-CSF) 6 mg with other doses for incidence of Grade 3-4 adverse event in dose-based analysis.

Comparison	Number of studies	Within-study bias	Reporting bias	Indirectness	Imprecision	Heterogeneity	Incoherence	Confidence rating
PEG-rhG-CSF Biosimilar 100 ug/kg:PEG-rhG-CSF Biosimilar 6 mg	1	No concerns	Low risk	No concerns	Major concerns	No concerns	No concerns	Moderate
PEG-rhG-CSF Biosimilar 13.2 mg:PEG-rhG-CSF Biosimilar 6 mg	1	Some concerns	Low risk	No concerns	Major concerns	No concerns	No concerns	Low
PEG-rhG-CSF Biosimilar 6 mg:PEG-rhG-CSF Biosimilar 7.5 mg	1	No concerns	Low risk	No concerns	Major concerns	No concerns	No concerns	Moderate
nonPEG-L 30 mg:PEG-rhG-CSF Biosimilar 6 mg	1	No concerns	Low risk	No concerns	Major concerns	No concerns	No concerns	Moderate
nonPEG-L 40 mg:PEG-rhG-CSF Biosimilar 6 mg	2	Some concerns	Low risk	No concerns	Major concerns	No concerns	No concerns	Low
nonPEG-L 50 mg:PEG-rhG-CSF Biosimilar 6 mg	2	Some concerns	Low risk	No concerns	Major concerns	No concerns	No concerns	Low
PEG-rhG-CSF Biosimilar 6 mg:rhG-CSF	4	No concerns	Low risk	No concerns	Major concerns	No concerns	No concerns	Moderate

13. Search Strategy

6 (#1 OR #2 OR #4) AND #3 AND #5

1 ("pegylate"[All Fields] OR "pegylated"[All Fields] OR "pegylates"[All Fields] OR "pegylating"[All Fields] OR "pegylation"[All Fields] OR "pegylations"[All Fields]) AND "granulocyte colony stimulating factor"[MeSH Terms]

2 pegfilgrastim[Supplementary Concept]

3 ("randomized controlled trial"[Publication Type] OR "controlled clinical trial"[Publication Type] OR "randomized"[Title/Abstract] OR "placebo"[Title/Abstract] OR "drug therapy"[MeSH Subheading] OR "randomly"[Title/Abstract] OR "trial"[Title/Abstract] OR "groups"[Title/Abstract]) NOT ("animals"[MeSH Terms] NOT "humans"[MeSH Terms])

4 bp$14[Title/Abstract] OR "da$3031"[Title/Abstract] OR "fulphila"[Title/Abstract] OR "g-lasta"[Title/Abstract] OR "HHPG-19K"[Title/Abstract] OR "ins$20"[Title/Abstract] OR "LA-EP2006"[Title/Abstract] OR "lapelga"[Title/Abstract] OR "lipegfilgrastim"[Title/Abstract] OR "lonquex"[Title/Abstract] OR "mecapegfilgrastim"[Title/Abstract] OR "myl$1401h"[Title/Abstract] OR "neulast*"[Title/Abstract] OR "Neulasta"[Title/Abstract] OR "nyvepria"[Title/Abstract] OR "peg filgrastim"[Title/Abstract] OR "pegfilgrastim"[Title/Abstract] OR "pegfilgrastim*"[Title/Abstract] OR "PEG-rhG-CSF"[Title/Abstract] OR "PEG-rmetHuG-CSF"[Title/Abstract] OR "PEGylated granulocyte colony stimulating"[Title/Abstract] OR "pegylated granulocyte colony-stimulating factor"[Title/Abstract] OR "pelmeg"[Title/Abstract] OR "r$1471"[Title/Abstract] OR "rgb$02"[Title/Abstract] OR "Ro 25-8315"[Title/Abstract] OR "sd$01"[Title/Abstract] OR "stimufend"[Title/Abstract] OR "udenyca"[Title/Abstract] OR "xm$22"[Title/Abstract]

5 breast neoplasms[MeSH Terms] OR "breast neoplasm"[Title/Abstract] OR "neoplasm breast"[Title/Abstract] OR "neoplasms breast"[Title/Abstract] OR "breast tumors"[Title/Abstract] OR "breast tumor"[Title/Abstract] OR "tumor breast"[Title/Abstract] OR "tumors breast"[Title/Abstract] OR "breast cancer"[Title/Abstract] OR "cancer breast"[Title/Abstract] OR "malignant tumor of breast"[Title/Abstract] OR "breast malignant tumor"[Title/Abstract] OR "breast malignant tumors"[Title/Abstract] OR "cancer of the breast"[Title/Abstract] OR "cancer of breast"[Title/Abstract] OR "malignant neoplasm of breast"[Title/Abstract] OR "breast malignant neoplasm"[Title/Abstract] OR "breast malignant neoplasms"[Title/Abstract] OR "mammary cancer"[Title/Abstract] OR "cancer mammary"[Title/Abstract] OR "cancer mammary"[Title/Abstract] OR "mammary cancers"[Title/Abstract] OR "mammary carcinoma human"[Title/Abstract] OR (("Carcinoma"[MeSH Terms] OR "Carcinoma"[All Fields] OR "Carcinomas"[All Fields] OR "carcinoma s"[All Fields]) AND "human mammary"[Title/Abstract]) OR (("Carcinoma"[MeSH Terms] OR "Carcinoma"[All Fields] OR "Carcinomas"[All Fields] OR "carcinoma s"[All Fields]) AND "human mammary"[Title/Abstract]) OR "human mammary carcinomas"[Title/Abstract] OR (("mammaries"[All Fields] OR "mammary glands, human"[MeSH Terms] OR ("Mammary"[All Fields] AND "glands"[All Fields] AND "Human"[All Fields]) OR "human mammary glands"[All Fields] OR "Mammary"[All Fields]

OR "Breast"[MeSH Terms] OR "Breast"[All Fields]) AND "carcinomas human"[Title/Abstract]) OR "human mammary carcinoma"[Title/Abstract] OR (("mammaries"[All Fields] OR "mammary glands, human"[MeSH Terms] OR ("Mammary"[All Fields] AND "glands"[All Fields] AND "Human"[All Fields]) OR "human mammary glands"[All Fields] OR "Mammary"[All Fields] OR "Breast"[MeSH Terms] OR "Breast"[All Fields]) AND "neoplasms human"[Title/Abstract]) OR (("human s"[All Fields] OR "humans"[MeSH Terms] OR "humans"[All Fields] OR "Human"[All Fields]) AND "mammary neoplasm"[Title/Abstract]) OR "human mammary neoplasms"[Title/Abstract] OR (("neoplasm s"[All Fields] OR "Neoplasms"[MeSH Terms] OR "Neoplasms"[All Fields] OR "Neoplasm"[All Fields]) AND "human mammary"[Title/Abstract]) OR (("neoplasm s"[All Fields] OR "Neoplasms"[MeSH Terms] OR "Neoplasms"[All Fields] OR "Neoplasm"[All Fields]) AND "human mammary"[Title/Abstract]) OR (("mammaries"[All Fields] OR "mammary glands, human"[MeSH Terms] OR ("Mammary"[All Fields] AND "glands"[All Fields] AND "Human"[All Fields]) OR "human mammary glands"[All Fields] OR "Mammary"[All Fields] OR "Breast"[MeSH Terms] OR "Breast"[All Fields]) AND "neoplasm human"[Title/Abstract]) OR "breast carcinoma"[Title/Abstract] OR "breast carcinomas"[Title/Abstract] OR "carcinoma breast"[Title/Abstract] OR "carcinomas breast"[Title/Abstract]

14. PRISMA checklist

Table. S2. PRISMA checklist.

PRISMA 2020 Checklist

Section and Topic	Item #	Checklist item	Location where item is reported
**TITLE**			
Title	1	Identify the report as a systematic review.	P7
**ABSTRACT**			
Abstract	2	See the PRISMA 2020 for Abstracts checklist.	P8
**INTRODUCTION**			
Rationale	3	Describe the rationale for the review in the context of existing knowledge.	P10-11
Objectives	4	Provide an explicit statement of the objective(s) or question(s) the review addresses.	P11
**METHODS**			
Eligibility criteria	5	Specify the inclusion and exclusion criteria for the review and how studies were grouped for the syntheses.	P12
Information sources	6	Specify all databases, registers, websites, organisations, reference lists and other sources searched or consulted to identify studies. Specify the date when each source was last searched or consulted.	P12
Search strategy	7	Present the full search strategies for all databases, registers and websites, including any filters and limits used.	P12
Selection process	8	Specify the methods used to decide whether a study met the inclusion criteria of the review, including how many reviewers screened each record and each report retrieved, whether they worked independently, and if applicable, details of automation tools used in the process.	P12
Data collection process	9	Specify the methods used to collect data from reports, including how many reviewers collected data from each report, whether they worked independently, any processes for obtaining or confirming data from study investigators, and if applicable, details of automation tools used in the process.	P12
Data items	10a	List and define all outcomes for which data were sought. Specify whether all results that were compatible with each outcome domain in each study were sought (e.g. for all measures, time points, analyses), and if not, the methods used to decide which results to collect.	P12
	10b	List and define all other variables for which data were sought (e.g. participant and intervention characteristics, funding sources). Describe any assumptions made about any missing or unclear information.	Supplementary material P49
Study risk of bias assessment	11	Specify the methods used to assess risk of bias in the included studies, including details of the tool(s) used, how many reviewers assessed each study and whether they worked independently, and if applicable, details of automation tools used in the process.	P12
Effect measures	12	Specify for each outcome the effect measure(s) (e.g. risk ratio, mean difference) used in the synthesis or presentation of results.	P12-13
Synthesis methods	13a	Describe the processes used to decide which studies were eligible for each synthesis (e.g. tabulating the study intervention characteristics and comparing against the planned groups for each synthesis (item #5)).	P13
	13b	Describe any methods required to prepare the data for presentation or synthesis, such as handling of missing summary statistics, or data conversions.	P13
	13c	Describe any methods used to tabulate or visually display results of individual studies and syntheses.	P13
	13d	Describe any methods used to synthesize results and provide a rationale for the choice(s). If meta-analysis was performed, describe the model(s), method(s) to identify the presence and extent of statistical heterogeneity, and software package(s) used.	P13
	13e	Describe any methods used to explore possible causes of heterogeneity among study results (e.g. subgroup analysis, meta-regression).	P13
	13f	Describe any sensitivity analyses conducted to assess robustness of the synthesized results.	P13
Reporting bias assessment	14	Describe any methods used to assess risk of bias due to missing results in a synthesis (arising from reporting biases).	P14

Section and Topic	Item #	Checklist item	Location where item is reported
Certainty assessment	15	Describe any methods used to assess certainty (or confidence) in the body of evidence for an outcome.	P14
**RESULTS**			
Study selection	16a	Describe the results of the search and selection process, from the number of records identified in the search to the number of studies included in the review, ideally using a flow diagram.	P14
	16b	Cite studies that might appear to meet the inclusion criteria, but which were excluded, and explain why they were excluded.	P14
Study characteristics	17	Cite each included study and present its characteristics.	Supplementary material P4
Risk of bias in studies	18	Present assessments of risk of bias for each included study.	P14-15
Results of individual studies	19	For all outcomes, present, for each study: (a) summary statistics for each group (where appropriate) and (b) an effect estimate and its precision (e.g. confidence/credible interval), ideally using structured tables or plots.	P15-17
Results of syntheses	20a	For each synthesis, briefly summarise the characteristics and risk of bias among contributing studies.	P14
	20b	Present results of all statistical syntheses conducted. If meta-analysis was done, present for each the summary estimate and its precision (e.g. confidence/credible interval) and measures of statistical heterogeneity. If comparing groups, describe the direction of the effect.	P15-17
	20c	Present results of all investigations of possible causes of heterogeneity among study results.	P15
	20d	Present results of all sensitivity analyses conducted to assess the robustness of the synthesized results.	P15
Reporting biases	21	Present assessments of risk of bias due to missing results (arising from reporting biases) for each synthesis assessed.	P15
Certainty of evidence	22	Present assessments of certainty (or confidence) in the body of evidence for each outcome assessed.	P15-17
**DISCUSSION**			
Discussion	23a	Provide a general interpretation of the results in the context of other evidence.	P18-20
	23b	Discuss any limitations of the evidence included in the review.	P20
	23c	Discuss any limitations of the review processes used.	P20
	23d	Discuss implications of the results for practice, policy, and future research.	P20
**OTHER INFORMATION**			
Registration and protocol	24a	Provide registration information for the review, including register name and registration number, or state that the review was not registered.	P12
	24b	Indicate where the review protocol can be accessed, or state that a protocol was not prepared.	P12
	24c	Describe and explain any amendments to information provided at registration or in the protocol.	-
Support	25	Describe sources of financial or non-financial support for the review, and the role of the funders or sponsors in the review.	P9
Competing interests	26	Declare any competing interests of review authors.	P9
Availability of	27	Report which of the following are publicly available and where they can be found: template data collection forms; data extracted from	-

Section and Topic	Item #	Checklist item	Location where item is reported
data, code and other materials		included studies; data used for all analyses; analytic code; any other materials used in the review.	

From: Page MJ, McKenzie JE, Bossuyt PM, Boutron I, Hoffmann TC, Mulrow CD, et al. The PRISMA 2020 statement: an updated guideline for reporting systematic reviews. BMJ 2021;372:n71. doi: 10.1136/bmj.n71

For more information, visit: http://www.prisma-statement.org/
